# Scaling Law of Urban Ride Sharing

**DOI:** 10.1038/srep42868

**Published:** 2017-03-06

**Authors:** R. Tachet, O. Sagarra, P. Santi, G. Resta, M. Szell, S. H. Strogatz, C. Ratti

**Affiliations:** 1 Senseable City Lab, Massachusetts Institute of Technology, Cambridge, MA 02139, USA; 2 Complexity Lab Barcelona, Universitat de Barcelona, 08028 Barcelona, SPAIN; 3 DRIBIA Data Research, 08012 Barcelona, SPAIN; 4 Istituto di Informatica e Telematica del CNR, 56124 Pisa, ITALY; 5 Hungarian Academy of Sciences, Centre for Social Sciences Országház utca 30, 1014 Budapest, HUNGARY; 6 Department of Mathematics, Cornell University, Ithaca, NY 14853, USA

## Abstract

Sharing rides could drastically improve the efficiency of car and taxi transportation. Unleashing such potential, however, requires understanding how urban parameters affect the fraction of individual trips that can be shared, a quantity that we call *shareability*. Using data on millions of taxi trips in New York City, San Francisco, Singapore, and Vienna, we compute the shareability curves for each city, and find that a natural rescaling collapses them onto a single, universal curve. We explain this scaling law theoretically with a simple model that predicts the potential for ride sharing in any city, using a few basic urban quantities and no adjustable parameters. Accurate extrapolations of this type will help planners, transportation companies, and society at large to shape a sustainable path for urban growth.

Mobility of people and goods has been vital to urban life since cities emerged more than 7,000 years ago^1^. Indeed, the success, prosperity, and livability of cities are directly related to the effectiveness of their mobility systems^2^. However, due to fixed schedules, limited coverage, and low quality of travel experience, public transportation systems accommodate only a fraction of the urban mobility demand^3^. The rest is satisfied by private vehicles and taxis, inefficient transportation modes that move only 1.3 passengers per vehicle on average^4^,^5^, causing the road congestion observed in most cities worldwide, with immense economic and societal costs^6^. Enhancing transportation efficiency is a key to rendering sustainable the urban growth predicted for the coming years^7^.

The emerging sharing economy^8^,^9^ promises to improve the efficiency of individual, on-demand transportation. Bridging the gap between shared but inflexible public transportation and flexible but not shared private transportation, novel services such as those provided by Uber^TM^, Lyft^TM^, and ZipCar^TM^ can significantly contribute to reducing road congestion and emissions. But the realizability of such potential benefits depends on the answer to a fundamental unsolved question: How compatible in space and time – and thus shareable – are individual mobility patterns?

While recent literature^10^,^11^,^12^,^13^,^14^,^15^ has unveiled spatial and temporal regularity of individual mobility patterns, very little is known about their mutual similarity. In a previous study^16^, we introduced the notion of a shareability network to quantify the spatial and temporal compatibility of individual trips. The nodes in the network represent trips, and links between them mark trips that can be shared. Two trips are defined to be shareable if they would incur a sharing delay of no more than *∆* minutes, relative to a single ride (see Supplementary Information). Let the shareability metric *S* denote the fraction of individual rides that can be shared. We found^16^ that taxi trips in New York City offer a shareability well above 95% for *∆* = 5 min, and that *S* increases rapidly with the number of trips available for sharing.

But that previous study^16^ left a key question unresolved: Might the results be peculiar to New York City? There was good reason to suspect so, given that New York is singular in several respects, namely, its large population, its small geographical area, and its enormous density of taxi traffic. In what follows, we study ride shareability in three other major world cities — San Francisco, Singapore, and Vienna — for which extensive data is available. Although these cities differ greatly from each other and from New York City in their traffic characteristics, population size, and geographical area, we find they all obey the same empirical law governing the potential for ride sharing. To the best of our knowledge, the existence of such a seemingly universal law has not been reported before. We explain the mechanism underlying this law of ride sharing using a simple mathematical model. The model’s prediction accounts for more than 90% of the variance in the data, and does so without any adjustable parameters. What is important here is the generality of the law, as well as its rapidly saturating shape, because together they imply that ride sharing could have a large beneficial impact in virtually any city, not just New York City.

## Results

**Figure 1:**
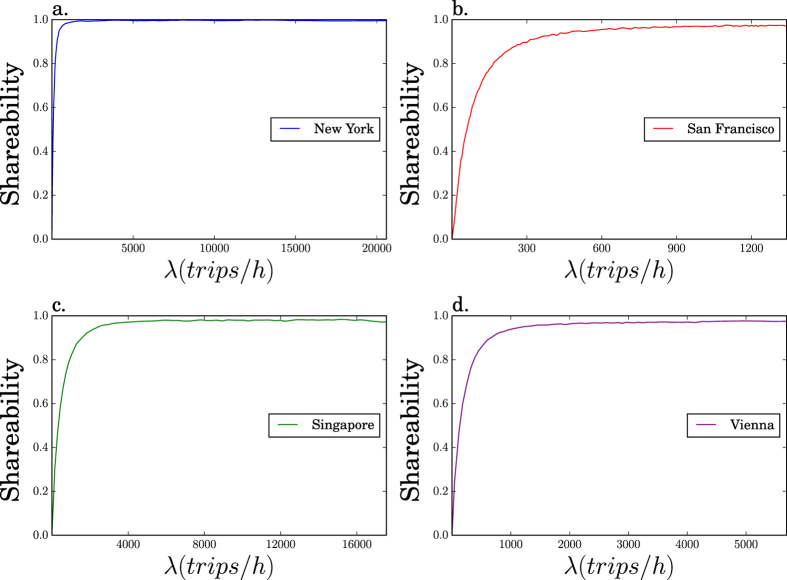
Shareability curves. The shareability curves for (**a**) New York, (**b**) San Francisco, (**c**) Singapore, and (**d**) Vienna. The curves were computed using a shareability network algorithm^16^ applied to data collected from over 156 million taxi trips in the four cities. See Methods for details about the datasets and algorithm.

Let *C* be a city, Ω(*C*) its spatial domain, |Ω(*C*)| its area, *v(C*) the average traffic speed in *C* and *λ* the average number of trips per hour with both endpoints in Ω(*C*). Figure 1(a) shows that the computed curve of shareability against *λ* for New York City^16^ closely resembles a “fast” saturation process, with a quick increase from lowest density, where shareability is minimal, to saturation where all trips can be shared. Our first main finding is that three other cities – San Francisco, Singapore, and Vienna (see Methods and Supplementary Information: Table S1a for datasets description and algorithms^17^) – show strikingly similar shareability curves (Fig. 1(b)–(d)). Such a similarity is remarkable, given that the shareability curves are obtained from data sets of real taxi trips, using a methodology that includes the hour-by-hour variability in traffic congestion (see Methods).

Each curve in Fig. 1 saturates rapidly as a function of *λ*. Their rapid saturation distinguishes them from other saturation phenomena observed in urban/geographical processes, such as the growth of retail locations^18^ and the spreading of innovations^19^, which are instead characterized by an initial “slow start” phase with a sigmoidal shape. Fast saturation of shareability is a plausible explanation for the great success of innovative ride and vehicle sharing apps such as UberPool^TM^, ZipCar^TM^, and Car2Go^TM^.

**Figure 2:**
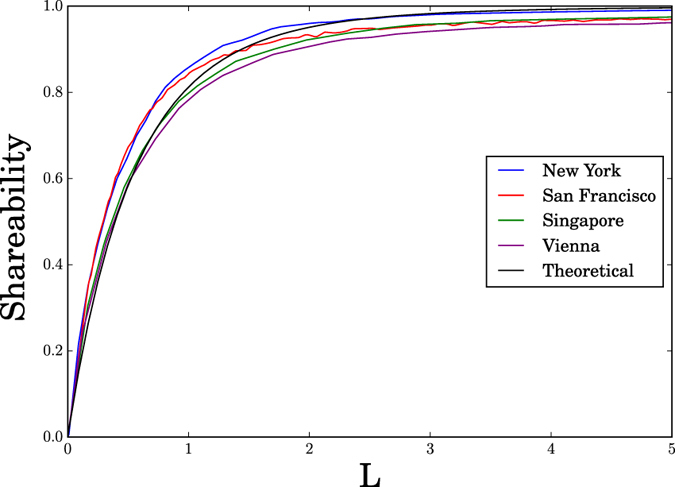
Shareability law. When replotted as functions of *L*, the computed shareability curves for New York, San Francisco, Singapore, and Vienna nearly coincide with each other and with the theoretical prediction given by Eq. (2). This rescaling involves no adjustable parameters.

The similarity we observe between cities actually goes beyond the resemblance of their shareability curves: a single linear rescaling of the *λ*-axis makes all the curves nearly coincident (Fig. 2; see also Methods and Supplementary Information: Table S2), suggesting that a common mechanism governs shareability in those four cities. The data collapse is achieved by replotting the computed shareability *S* versus the dimensionless quantity


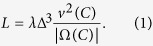


The greater the *L*, the greater the shareability.

The quality of the data collapse indicates that a few urban level parameters, when combined into the dimensionless group *L*, suffice to accurately model a complex quantity like the fraction of trips that can be shared in a city. This result is all the more surprising when one considers that *L* is defined in terms of the *average* daily traffic speed in the city, while the shareability curves have been derived using hourly, street-level traffic speed estimations. Evidently, the variability of congestion occurring at different times of day has a limited effect on our predictions. We do not ignore that variability; on the contrary, it is captured by the data we use to derive the shareability curves. Yet the fact that our simple model accounts so well for those shareability curves demonstrates that the variability is not a dominant effect. The universal curve can be explained by using a single, average value of the traffic velocity *v*.

The particular combination of urban parameters in Eq. (1) can be rationalized by dimensional analysis. Intuitively, *L* represents a ratio between two timescales: the sharing delay *∆* and the characteristic waiting time *t*_wait_ for a trip to be generated in a user’s vicinity. To see this, imagine that you are looking for a cab. Since λ is the average rate at which taxi trips are generated, 1/λ is the characteristic time for a new trip to be generated, somewhere in the city. But the city as a whole is not what concerns you. What matters more is how long you can expect to wait for a new trip to be generated in your vicinity. The characteristic linear scale of a vicinity is *v*Δ, the distance a cab moving at speed *v* would travel in the delay time Δ that another passenger could tolerate. Since the city has a total area |Ω| and each vicinity has area (*v*Δ)^2^, there are about |Ω|/(*v*Δ)^2^ vicinities in total. Assuming that trips are generated uniformly in space, you would expect a trip to be generated in your vicinity every 
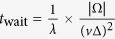
 time units. Hence the ratio of the tolerable delay time Δ to the expected waiting time is Δ/*t*_wait_ = λ*v*^2^Δ^3^/|Ω| = *L*.

At a more refined level, the influence of urban parameters on shareability can be approached mathematically as follows. Intuitively, one expects that shareability should be positively related to *∆, v(C*), and *λ*. Indeed, as *∆* and *v(C*) increase, people become more tolerant about sharing delay and a larger urban space can be covered without exceeding the delay^16^. The effect of increasing trip density is more complex to assess since it simultaneously introduces new rides and new ride-sharing opportunities. However, the additional trips are drawn from the same distribution as the original ones, so they possess similar spatiotemporal properties, which on average results in an increase of shareability as a function of *λ*.

Assuming that rides are generated independently in the city according to a given spatiotemporal distribution, we wish to compute the probability that a ride can be shared as a function of *∆, v(C*), and *λ*. Tackling this problem directly is very difficult, since the probability of actually sharing a ride depends not only on the spatiotemporal availability of candidate trips to share, but also on how potentially shareable trips are paired together, which in turn depends on complex structural properties of the underlying shareability network. Nevertheless, the spatial dimension of the problem, coupled with the observed fast saturation of the shareability curve, suggest analogies with geometric random graphs^20^ and percolation theory^21^. A common trait of these theories is that complex network structural properties such as connectivity can be closely approximated by much simpler properties, such as the existence of isolated nodes. This turns out to be the case also for shareability networks; we find that shareability *S* is highly correlated with the number of isolated nodes in the shareability network (Methods).

Based on the above discussion, we can model shareability by fixing an arbitrary trip *T* and estimating the probability that there exists at least one other trip *T*′ shareable with *T*. More specifically, an arbitrary trip *T* starting at time *t*_*0*_ and going from origin *o* to destination *d* defines a trajectory in space and time. For fixed *∆* and average traffic speed *v(C*), we define the notion of the shareability shadow *s(T*) surrounding *T* and confining the region of sharing opportunities (Supplementary Information: Figs S1 and S2). For another trip *T*′ to be shareable with *T*, its trajectory needs to overlap (i.e., to take place at the same time, at least partially) and to be “aligned” (i.e., not deviate too much direction-wise) with *s(T*). Those two conditions simply translate our upper bound *∆* on delays into a geometric condition stating that shareable trips should be close enough in terms of trajectories, where close enough is quantified through the volume of *s(T*) chosen depending on *v(C*) and *∆*. Analytically, the expected shareability becomes the probability that a compatible trip will be generated in the shareability shadow (see Supplementary Information: “Supplementary Equations”). To compute that quantity, the previously mentioned spatiotemporal distribution of trips has to be determined. Among the different options we considered, the following one gave the best compromise between accuracy and tractability: origin point *o* chosen uniformly in Ω, and destination point *d* chosen uniformly in a disk centered on *o* of radius *R* (ignoring boundary effects for the sake of simplicity). The geometry of the city plays a minimal part in the definition, which allows us to derive analytical formulas for the shareability. For *R* large enough, we find that *S* becomes independent of *R*, and the city’s influence on the shareability only appears through the quantity *L*. We prove that (see Supplementary Information: “Supplementary Equations”)


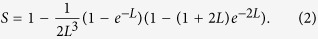


We tested our model predictions on the four cities mentioned above and found a strong agreement with the respective shareability curves (Supplementary Information: Fig. S3), with *R*^*2*^ values ranging from 0.91 to 0.98.

## Discussion

Our main contribution is the discovery of a unifying mathematical law that governs the potential for ride sharing in cities of diverse sizes and traffic characteristics. We have also proposed a simple model that accounts for the law, and does so with no adjustable parameters. The fidelity of the model suggests that the mechanisms governing ride sharing, in a real-world scenario where trips are performed on a road network with traffic congestion, can be accurately characterized by a model built on such simplifying assumptions as Euclidean geometry, straight-line trajectories, and extremely basic shareability shadow shapes. In particular, most of the knowledge required to determine shareability is contained in the dimensionless group *L*. Being relatively easy to estimate, and the only quantity required for our (otherwise parameter-free) framework, *L* gives the model strong predictive power.

A final important feature of the framework is its flexibility, which allows for potential enhancements. Relaxing some of the model’s underlying assumptions might produce even greater accuracy. In particular, the shape of the shareability shadows could be modeled using conics. Or more realistic origin-destination patterns could be considered, but this would be done at the expense of closed-form formulas for shareability; the four-dimensional integral representing it (Supplementary Information: “Supplementary Equations”) would then require stochastic approximations to be computed (using the VEGAS algorithm, for instance^22^). On a potentially more impactful note, if the exact effect of congestion on average speed were known, through modeling or pervasive sensors, the model could be extended to take the following second-order effect into account: ride sharing reduces congestion and increases average travel speed, thereby increasing shareability (see Supplementary Information: “Supplementary Equations”).

The in-depth study of the cities and their curves (Fig. 2 and S3) shows a few interesting features. New York City cabs live up to their reputation with a shareability curve reaching over 99% compared to ~97% for the other three cities. The very high and homogeneous density of people in Manhattan (the only borough of New York City included in our study) might explain this difference between cities. For San Francisco, Singapore and Vienna, the entire city, including more sparsely populated areas, was considered. It is possible that those areas’ “outlier” trips create small discrepancies between shareability curves. Large uninhabited places (e.g., Lainzer-Tiergarten in Vienna, and Singapore’s central water catchment) were taken into account while computing the cities’ areas, which might also explain certain differences between them.

Our findings quantify the effects of ride sharing on the urban environment and shed light on the recent upheaval that ride sharing has caused in cities worldwide. Furthermore, they offer valuable guidance towards designing more efficient mobility systems in the future. Tables 1 and 2 show the urban parameters and corresponding predictions for ride shareability in several major world cities. Even for low trip density, and allowing delays no longer than Δ = 5 minutes, the potential for sharing is massive.

## Methods

The New York dataset has been obtained from the New York Taxi and Limousine Commission for the year 2011 via a Freedom of Information Act request. It is the same as the dataset used in refs 16 and 17. The San Francisco dataset is freely available^23^. The Vienna and Singapore datasets were provided to the MIT SENSEable City Lab by AIT and the Singapore government, respectively.

The New York dataset spans more than an entire year and contains all taxi trips generated in the area of New York by its approximately 13,500 taxis. The other datasets span roughly over a month and contain records provided by a single taxi operator. The total number of cabs in San Francisco is officially 1,494^24^, and the number of taxis tracked in the data set is about 500. For Singapore, the official figure is 25,176^25^, and our dataset refers to about 16,000 taxis. For Vienna, we have traces of about 1,000 taxis, while the total number of taxis operating in the city is unknown. Dataset details are reported in the Supplementary Information, Table S1.

We have applied the same filtering procedure to all the datasets: only trips performed while a customer occupied the taxi were considered in the analysis. From these trips, we only kept the ones with start and end GPS positions within 200 meters of the closest intersection present in the considered area of study. Such an area was obtained by considering the borough of Manhattan (NY), the entire island of Singapore (SI), and both the urban areas of San Francisco (SF) and Vienna (VI), including the road to the airport. We included the airports of San Francisco, Singapore, and Vienna since trips to and from them account for a substantial fraction of the dataset.

The intersections were obtained from Open Street Map^26^ (see Supplementary Information, Fig. S4), considering only primary and secondary level roads and by manually merging all repeated elements corresponding to every given intersection (using GQIS^27^). All trip coordinates were provided in longitude-latitude pairs using the *WGS84* ellipsoid but have been projected to Euclidean UTM coordinates using the zones specified in the Supplementary Information, Table S1a.

After pre-processing, each trip is uniquely identified by a tuple containing starting and ending (*latitude, longitude*) coordinates, which correspond to the coordinates of the intersections closest to start and ending coordinates of a trip, and by a pickup and dropoff time. Pickup and dropoff times are used to estimate travel times between any two intersections in the city for each of the 24 hours, according to the procedure described in ref. 16. This method allows accounting for the effect of traffic on travel time when computing the shareability networks used to obtain the shareability curves shown in the paper. Shareability networks for the four cities were obtained using the method described in ref. 16.

To generate the saturation curves used in the paper, two procedures were required. For the New York, San Francisco and Singapore datasets, for which the shareability curves are saturated, the lower parts of the curves (corresponding to small trip densities *λ*) were obtained by randomly and uniformly subsampling the database of actual trips up to the desired density. For the Vienna case, a second procedure was necessary to reach densities higher than those in the dataset (explaining why Vienna curves show *λ* and *L* values larger than the *λ*_*f*_ and *L(C*) from Table S1b reported in the Supplementary Information). We call that procedure *supersampling*, and extend an existing method^17^.

The above procedure is used to interpolate trips from a given sample in a static manner in time. It is based on inferring a city’s invariant collection of transition probabilities {*p*_*ij*_}, where *ij* enumerates all possible intersection pairs. Such a collection of values is normalized (Σ_*ij*_*p*_*ij*_ = 1) and represents the probability that a given trip is generated at intersection *i* and ends at intersection *j*. Such a collection is shown^17^ to be extremely stable in time, and a procedure is developed to infer the complete set of values (note that in general *p*_*ij*_ ≠ 0 for all *i* and *j*). Once this collection of values is obtained, for a given density (total number of trips *T*_*trips*_ generated in a given timespan *τ*) the allocation of trips to each intersection pair *ij* reads <*t*_*ij*_> = *T*_*trips*_ *p*_*ij*_ with *t*_*ij*_ an integer random variable following a Poisson distribution.

We have extended the method of ref. 7 to allow for *dynamic supersampling* in time. Algorithm 1 (see Supplementary Information, Algorithm S1) exploits the exponential nature of inter-events times between trips (see Supplementary Information, Fig. S5) coupled with the statistics of daily and hourly trip generation (see Supplementary Information, Fig. S6). For every day, the algorithm distributes the empirical number of daily generated trips *T*_*d*_ over hourly intervals according to the empirical probability *q*_*h*_ = *Ť*_*h*_/Σ_*ĥ*_ *Ť*_*ĥ*_ (where *Ť*_*h*_ is the average number of trips observed during hour *h* and 0 ≤ *h, ĥ* ≤ 23) and then distributes the generated trips over intersections according to *p*_*ij*_. Finally, for each intersection, the number of allocated trips is distributed in time according to a Poisson process. The code for this extension was made public^28^.

## Additional Information

**How to cite this article**: Tachet, R. *et al*. Scaling Law of Urban Ride Sharing. *Sci. Rep.* **7**, 42868; doi: 10.1038/srep42868 (2017).

**Publisher's note:** Springer Nature remains neutral with regard to jurisdictional claims in published maps and institutional affiliations.

## Supplementary Material

Supplementary Information

## Figures and Tables

**Table 1:** City Parameters.

City Parameters								
Cities	Amsterdam	Berlin	London	Newcastle	Paris	Prague	Rome	Santiago
Area (*km*^*2*^)	219	892	1572	360	105	496	1285	641
Speed (*km/h*)	34	19	19	42	31	37	30	31

Area (*km*^2^) and average speed (*km/h*) of vehicles in different cities around the world^29^,^30^.

**Table 2:** Shareability in different world cities.

Shareability									
Cities		Amsterdam	Berlin	London	Newcastle	Paris	Prague	Rome	Santiago
*Trips/h/km*^2^	0.5	45%	17%	17%	59%	39%	51%	37%	39%
	2.5	92%	60%	60%	97%	89%	95%	88%	89%
	4.5	98%	80%	80%	99%	97%	99%	97%	97%
	6.5	99%	89%	89%	100%	99%	100%	99%	99%
	8.5	100%	94%	94%	100%	100%	100%	99%	100%

Shareability as a function of spatiotemporal trip density, measured in units of trips per hour per square kilometer. For comparison, the spatiotemporal trip densities of taxi rides in our datasets are: 344.12 *trips/h/km*^*2*^ in New York, 24.46 in Singapore, 12.63 in San Francisco and 0.95 in Vienna (Supplementary Information Table S1b), generating a shareability of almost 100% for the first three and of 83% for Vienna.
